# Dexamethasone and dexmedetomidine as adjuvants to ropivacaine do not prolong analgesia in wound infiltration for lumbar spinal fusion: a prospective randomized controlled study

**DOI:** 10.1186/s13018-023-04145-1

**Published:** 2023-09-04

**Authors:** Wenkai Li, Khan Akhtar Ali, Xinyue Deng, Yong Li, Zhong Fang

**Affiliations:** Present Address: Department of Orthopedics, Tongji Hospital, Tongji Medical College, Huazhong University of Science and Technology, Wuhan, 430030 China

**Keywords:** Transforaminal lumbar interbody fusion (TLIF), Local anesthetics, Dexamethasone, Dexmedetomidine

## Abstract

**Background and objectives:**

Local anesthetics (LAs) are widely used to infiltrate into surgical wounds for postoperative analgesia. Different adjuvants like dexamethasone and dexmedetomidine, when added to LA agents, could improve and prolong analgesia. The aim of this trial was to evaluate the analgesic efficacy and opioid-sparing properties of dexamethasone and dexmedetomidine when added to ropivacaine for wound infiltration in transforaminal lumbar interbody fusion (TLIF).

**Methods:**

We conducted a controlled study among 68 adult patients undergoing TLIF, which was prospective, randomized and double-blind in nature. The participants were divided into four equal groups at random. Group R was given 150 mg of 1% ropivacaine (15 mL) and 15 mL of normal saline. Group R + DXM received 150 mg of 1% ropivacaine (15 mL) and 10 mg of dexamethasone (15 mL). Group R + DEX received 150 mg of 1% ropivacaine (15 mL) and 1 µg/kg of dexmedetomidine (15 mL). Lastly, group R + DXM + DEX was given 150 mg of 1% ropivacaine (15 mL), 10 mg of dexamethasone and 1 µg/kg of dexmedetomidine (15 mL). The primary focus was on the length of pain relief provided. Additionally, secondary evaluations included the amount of hydromorphone taken after surgery, the numerical rating scale and safety assessments within 48 h after the operation.

**Results:**

Based on the *p* value (*P *> 0.05), there was no significant variance in the duration of pain relief or the total usage of hydromorphone after surgery across the four groups. Similarly, the numerical rating scale scores at rest and during activity at 6-, 12-, 24- and 48-h post-surgery for all four groups showed no difference (*P *> 0.05). However, the incidence of delayed anesthesia recovery was slightly higher in group R + DEX and group R + DXM + DEX when compared to group R or group R + DXM. Furthermore, there were no significant differences between the four groups in terms of vomiting, nausea, dizziness or delayed anesthesia recovery.

**Conclusion:**

For wound infiltration in TLIF, the addition of dexamethasone and dexmedetomidine to ropivacaine did not result in any clinically significant reduction in pain or opioid consumption and could prompt some side effects.

## Introduction

Managing postoperative pain in spine surgery patients is a challenging task. The surgery often results in moderate to severe pain, which can lead to poor surgical outcomes, delayed recovery, increased complications and low patient satisfaction. To address this issue, multimodal analgesia (MMA) is becoming more commonly used in spine surgery, as there is a growing body of evidence supporting its effectiveness. There are various methods used in MMA for pain management, such as preemptive analgesia, nonsteroidal anti-inflammatory drugs (NSAIDs), acetaminophen, neuromodulator drugs, local anesthetic (LA) infiltration and fascial compartment blocks [1, 2]. MMA regimens acting through different mechanisms have been developed as a means of improving postoperative pain management and offer significant opioid sparing and minimize the side effects of individual drugs [3]. One particularly intriguing method for pain management after surgery is the use of local anesthetics (LA) which are injected directly into the subcutaneous tissue or surgical wound. Studies have shown that LA infiltration is a straightforward, safe and effective way to alleviate postoperative pain for joint, abdominal, tonsillar and spine surgeries [3–6]. Using LA infiltration alone may not be as effective as nerve axis or peripheral nerve block. To increase its effectiveness, adjunct drugs such as opioids, NSAIDs, steroids, alpha-2 agonists, ketamine, magnesium, neo-saxitoxin and methylene blue are added to prolong its duration. Thirteen RCTs have investigated using dexmedetomidine as an adjunct for various surgeries [4]. Two adjuncts that have proved effective in improving postoperative analgesia when used with LA are dexmedetomidine and dexamethasone. Dexmedetomidine is an alpha-2 adrenoreceptor agonist that is non-selective. When administered perineurally, it is believed to keep Aδ(delta) and C neurons in a hyperpolarized state, which inhibits the generation of action potentials [5, 7, 8]. The dosages used ranged from 0.5 to 5 µg/kg. When dexmedetomidine was combined with LA, it resulted in reduced opioid requirements post-operation, longer-lasting pain relief and lower pain scores after the surgery [9–13]. Dexamethasone is a potent glucocorticoid that stimulates receptors on neuronal membranes, reducing excitability of unmyelinated C fibers. It may also induce vasoconstriction or systemic anti-inflammatory processes [5]. Two high-quality studies which added dexamethasone to LA infiltration for laparoscopic cholecystectomy and cesarean section [14] proved that dexamethasone with LA had a mild analgesic benefit compared with LA alone. The addition of dexamethasone to LA for brachial plexus block will increase the block duration, depending on the type of LA, by ~ 2–3 h when added to a medium acting LA, and up to 10 h when added to a long-acting drug [15]. Due to the different mechanisms of action, we hypothesized that added dexamethasone and dexmedetomidine to LA could further prolong the duration and enhance the efficacy. In addition, the benefits of combining the two drugs with LA for wound infiltration in spine surgery are not yet conclusively proved. We conducted a study to determine whether adding two drugs to ropivacaine for wound infiltration would increase the duration of the block and improve postoperative pain relief. The study was prospective, randomized, double-blind and controlled. We compared the effectiveness of using each drug as an adjunct alone with ropivacaine.

## Method

See Fig. 1.

**Fig. 1:**
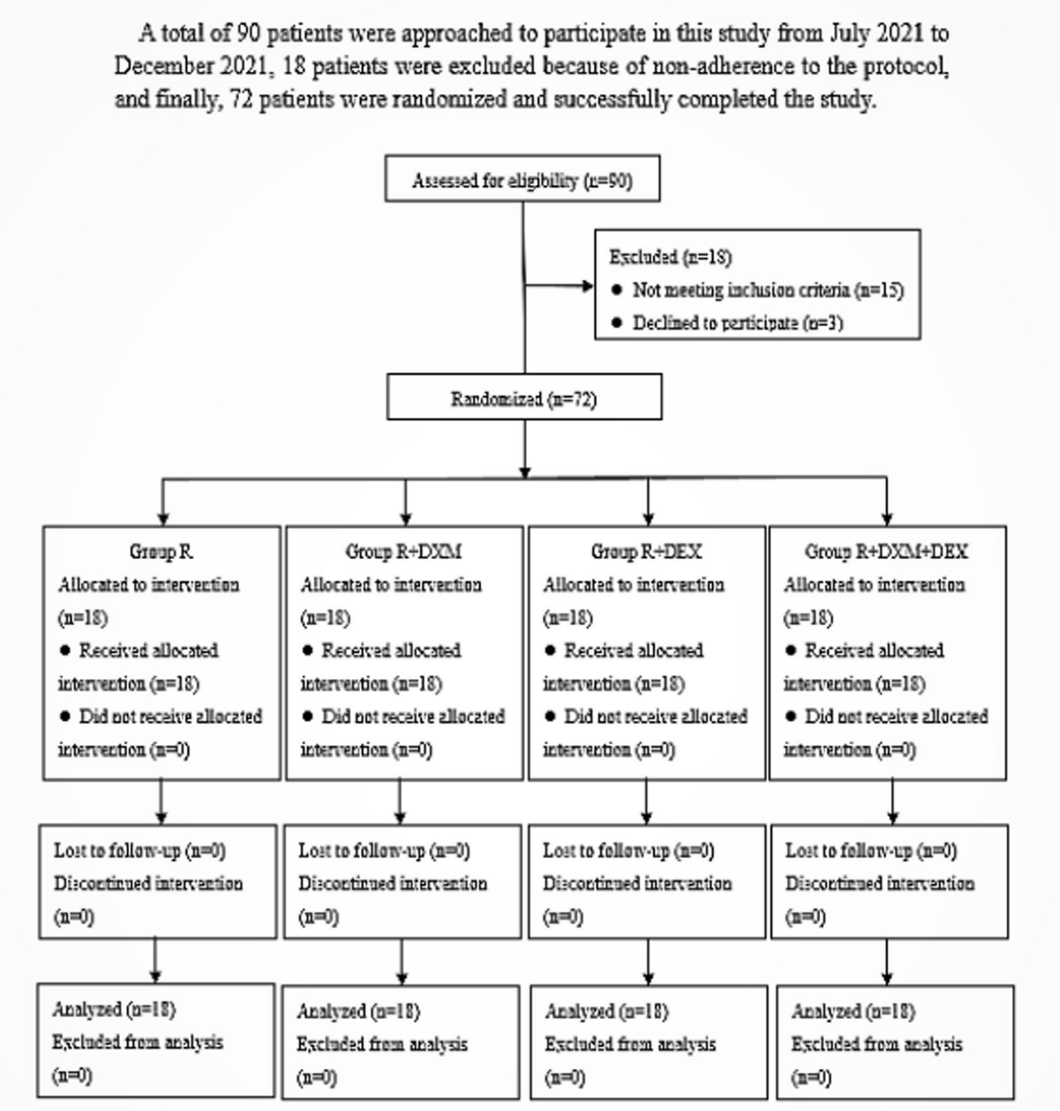
Study consort chart

### Trial design and participants

We carried out a single-center, prospective, randomized, double-blind, controlled trial with institutional ethics committee approval in which patients with lumbar radiculopathy due to disk herniation or lumbar spinal stenosis were assigned to receive transforaminal lumbar interbody fusion (TLIF). Informed consent was obtained from all participants before data collection. This study was registered in the Chinese Clinical Trials Registry under registration number ChiCTR2100042880. We enrolled patients between 30 and 75 years of age who met the inclusion and exclusion criteria of the study (Table 1).

**Table 1:** Inclusion and exclusion criteria

Inclusion criteria	Exclusion criteria
1. Low back pain and/or leg pain, and MRI confirmed central canal stenosis, or lateral recess stenosis or intervertebral foramen stenosis of lumbar vertebra 2. Segments of lumbar spinal fusion < 3 3. Duration of symptoms and conservative treatment > 3 months 4. American Society of Anesthesiologists (ASA) physical status classes I and II 5. Patient-controlled analgesia device (PCA) can be used independently	1. Previous lumbar spine surgery 2. Myelopathy, cauda equine syndrome or other spinal conditions (cancer, rheumatologic disorders, neurologic disorders) 3. Ischemic heart disease; bradycardia (heart rate < 50 beats/min) with or without cardiac conduction or arrhythmia; diabetes mellitus; severe osteoporosis; neuromuscular and endocrine diseases; coagulation disorders; adrenoreceptor agonist or antagonist therapy; allergy to the study drugs 4. Recent or previous history of alcohol, opioid or narcotic drug dependence 5. Chronic pain syndromes 6. Pregnant or lactating women

### Randomization and blinding

Participants who gave their consent for the study were randomly assigned to one of four groups using a computer-generated list of random numbers with a 1:1:1:1 ratio. Before the incision was closed, 30 ml of study drugs were injected into the skin, subcutaneous tissue and paraspinal muscle.

Here are the different groups and their respective solutions:

1. Group R: 15 mL of ropivacaine 1% mixed with 15 mL of NS (normal saline).
2. Group R + DXM: 15 mL of ropivacaine 1% mixed with 10 mg of dexamethasone in 15 mL NS.
3. Group R + DEX: 15 mL of ropivacaine 1% mixed with 1 µg/kg of dexmedetomidine in 15 mL NS.
4. Group R + DXM + DEX: 15 mL of ropivacaine 1% mixed with 10 mg of dexamethasone and perineural dexmedetomidine 1 µg/kg in 15 mL NS.

All patients did not take dexmedetomidine or dexamethasone before and after surgery. The randomization tables were kept in the hospital pharmacy, and a nurse who was not involved in the study prepared the study medication and the patient-controlled anesthesia (PCA) solution. The group assignments were kept blinded from the patients, surgeons, anesthesiologists and researchers who collected the data.

### Interventions

Prior to the study procedure, the patients were given instruction on how to operate the PCA pump and assess their pain using the numerical rating scale (NRS). Once they entered the operating room, a solution of Ringer's lactate was infused via an 18-Gauge intravenous cannula that had been placed in a peripheral vein on their forearm. During the procedure, various vital signs were continuously monitored, including ECG, heart rate, invasive blood pressure, respiratory rate and pulse oxygen saturation. To establish general anesthesia, a combination of 0.30 mg/kg of etomidate, 0.4 mg/kg of sufentanil and 0.2 mg/kg of cisatracurium was administered. Propofol, sevoflurane, remifentanil and cisatracurium were used to maintain anesthesia throughout the procedure. All surgical procedure was performed by the same surgeon. After the surgery, the surgeon injected 15 mL into each side of the surgical incision for wound infiltration. All patients were given PCA for postoperative pain relief. The PCA protocol included hydromorphone hydrochloride (10 mg), tropisetron hydrochloride (5 mg) and normal saline (85 ml). The PCA was programmed to administer a 1 mL bolus with a lockout time interval of 10 min and no baseline infusion. The PCA was given when the patient reported a pain score of ≥ 4 on the NRS or requested it.

### Outcomes

The main focus of our study was to measure the length of time before the first request for pain relief, known as the duration of analgesia. Additionally, we also recorded the total use of hydromorphone hydrochloride in patient-controlled analgesia (PCA) within 48 h, as well as pain scores measured at rest and during activity at six different time points following surgery (6, 12, 24, 36 and 48 h). It is important to note that we only collected data on pain associated with the surgical wound, and not any nerve-related symptoms. After the surgery, any negative effects like bradycardia (heart rate lower than 50 beats per minute), hypoxemia (oxygen saturation below 90%), hypotension (systolic blood pressure lower than 90 mmHg or a decrease of more than 20% from baseline), respiratory depression (less than 10 breaths per minute for over 10 min), headache, itchiness, nausea, vomiting and neurotoxicity were noted and addressed within 48 h post-surgery. Hypotension is treated by fluid loading, intravenous ephedrine or phenylephrine. Atropine was used to treat bradycardia. Respiratory depression was treated with oxygen or naloxone until RR ≥ 15 times per minute.

### Statistical analysis

To determine the sample size needed for our study, we used PASS V.11.0 (PASS, NCSS, USA). Our primary focus was on measuring the duration of analgesia. In our pre-trial study of twelve patients (three in each group), the mean duration of analgesia in R, R + DXM, R + DEX and R + DXM + DEX group were 663 ± 261 min, 636 ± 373 min, 1120 ± 330 min and 880 ± 302 min, respectively. At a power of 0.80 and an alpha error of 0.05, the required sample size for each group was calculated to be 15. Considering the dropouts and incomplete follow-up, 18 patients per group and a total of 72 patients were suggested for this study.

Statistical analysis was performed with SPSS V.19.0 for Windows. The distribution of the data was first evaluated for normality using the Kolmogorov–Smirnov test. Normally distributed continuous variables are expressed as the means ± standard deviation (SD). For duration of analgesia, total postoperative hydromorphone hydrochloride consumption and NRS data, ANOVA followed by the Bonferroni post hoc test was used. Categorical data (the incidence of side effects) were presented as frequencies (%) and analyzed using Fisher’s exact test. *P *< 0.05 value was considered to be statistically significant.

## Results

**Fig. 2:**
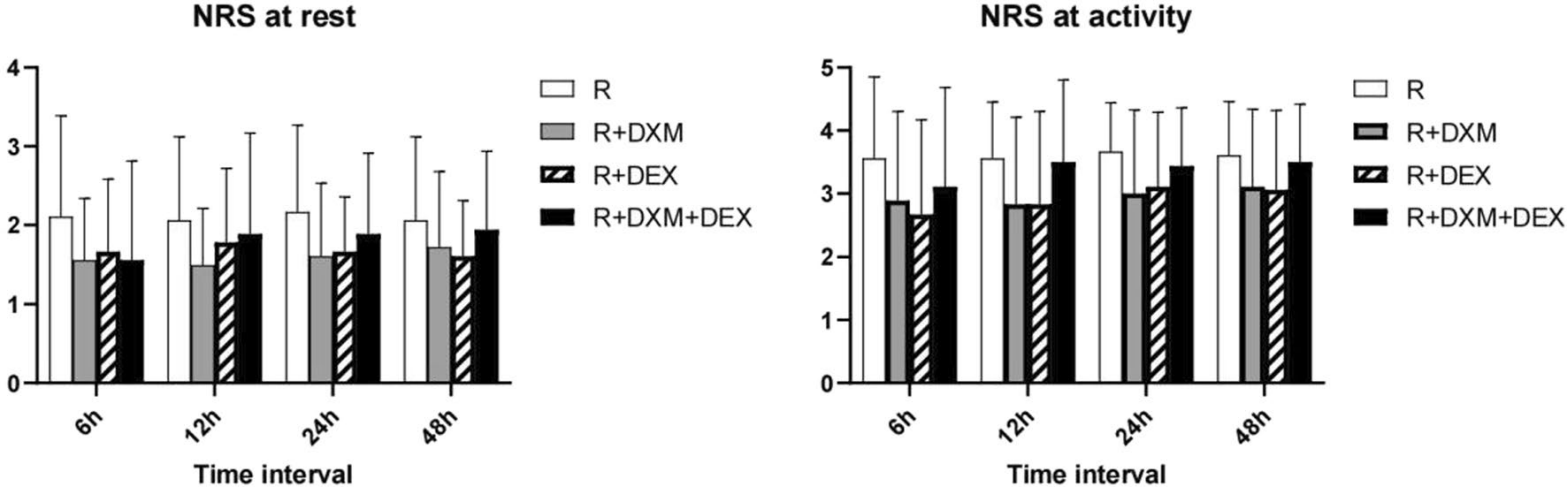
Postoperative pain severity NRS score at rest and activity at 6, 12, 24 and 48 h postoperatively. R: ropivacaine; R + DXM: ropivacaine + dexamethasone; R + DEX: ropivacaine + dexmedetomidine; R + DXM + DEX: ropivacaine + dexamethasone + dexmedetomidine. NRS, numerical rating scale

This study included 90 patients from July to December 2021. However, 18 patients were excluded due to non-compliance with the protocol. Ultimately, 72 patients were randomly selected and completed the study successfully (refer to Figure 1). There was no significant difference among the four groups in duration of analgesia and total postoperative hydromorphone consumption (*P *> 0.05; Table 2). The groups R + DEX and R + DXM + DEX experienced more delays in anesthesia recovery compared to groups R or R + DXM. However, there were no significant differences in the occurrences of vomiting, nausea or dizziness among the four groups. Additionally, there were no cases of bradycardia, hypotension, hypoxemia, respiratory depression, neurotoxicity or pruritus recorded, as shown in Table 3. There were no significant differences in demographic data and surgical characteristics among the four groups (Table 4; *P *> 0.05). There was no notable distinction between the four groups in terms of their postoperative pain severity NRS score while at rest or during activity at 6, 12, 24 and 48 h following the surgery (with a *P* value greater than 0.05 as shown in Fig. 2).

**Table 2:** Postoperative analgesic data in the four groups

	Group R	Group R + DXM	Group R + DEX	Group R + DXM + DEX	*P* value
Duration of analgesia (min)	550.00 ± 270.10	600.22 ± 398.25	767.22 ± 436.86	743.89 ± 389.07	0.242
Total hydromorphone consumption (mg)	2.49 ± 1.88	2.34 ± 2.03	2.83 ± 1.61	2.36 ± 1.38	0.819

A *p* value < 0.05 was considered statistically significant

*R* Ropivacaine, *R* + *DXM* ropivacaine and dexamethasone, *R* + *DEX* ropivacaine and dexmedetomidine, *R* + *DXM* + *DEX* ropivacaine, dexamethasone and dexmedetomidine

**Table 3:** Incidence of side effects

Side effects	Group R	Group R + DXM	Group R + DEX	Group R + DXM + DEX	*P* value
Bradycardia	0 (0)	0 (0)	0 (0)	0 (0)	–
Hypotension	0 (0)	0 (0)	0 (0)	0 (0)	–
Hypoxemia	0 (0)	0 (0)	0 (0)	0 (0)	–
Respiratory depression	0 (0)	0 (0)	0 (0)	0 (0)	–
Pruritus	0 (0)	0 (0)	0 (0)	0 (0)	–
Neurotoxicity	0 (0)	0 (0)	0 (0)	0 (0)	–
Nausea/vomiting	1 (0)	0 (0)	2 (11.1)	2 (11.1)	0.237
Dizziness	0 (0)	0 (0)	1 (5.6)	1 (5.6)	0.561
Delayed anesthesia recovery	0 (0)	0 (0)	4 (22.2)	3 (16.7)	0.045

A *p* value < 0.05 was considered statistically significant

*R* Ropivacaine, *R* + *DXM* ropivacaine and dexamethasone, *R* + *DEX* ropivacaine and dexmedetomidine, *R* + *DXM* + *DEX* ropivacaine, dexamethasone and dexmedetomidine

**Table 4:** Demographic data and surgical characteristicss

	Group R	Group R + DXM	Group R + DEX	Group R + DXM + DEX	*P* value
Age (years)	55.06 ± 10.70	56.28 ± 5.77	54.11 ± 7.10	56.44 ± 8.70	0.386
BMI (kg/m^2^)	23.22 ± 3.27	23.91 ± 3.79	24.87 ± 3.24	23.24 ± 3.20	0.422
Gender (male/female)	9:9	6:12	10:8	7:11	0.522
ASA class (I/II)	3:15	1:17	2:16	2:16	0.771
aCCI	1.33 ± 1.09	1.28 ± 0.75	1.00 ± 1.03	1.44 ± 0.92	0.378
Duration of surgery (hour)	4.28 ± 1.02	4.06 ± 0.54	3.78 ± 1.26	4.31 ± 1.05	0.485
Fusion levels (1/2)	11:7	12:6	12:6	12:6	0.980

*aCCI* Age-adjusted Charlson Comorbidity Index, *BMI* body mass index, *ASA* American Society of Anesthesiologists, *R* ropivacaine, *R* + *DXM* ropivacaine and dexamethasone, *R* + *DEX* ropivacaine and dexmedetomidine, *R* + *DXM* + *DEX* ropivacaine, dexamethasone and dexmedetomidine

A *p* value < 0.05 was considered statistically significant

## Discussion

After spinal surgeries, opioids have been traditionally used as the primary pain reliever despite their negative side effects. However, there has been a rising trend in utilizing multimodal analgesia (MMA) regimens. A study conducted by John S Jones et al. found that there was no significant difference in opioid use with the use of dexmedetomidine on any postoperative day. Moreover, the use of continuous local anesthetic infusions instead of dexmedetomidine can offer effective pain control, ensure safety, reduce postoperative hospital stays and eliminate the need for ICU admissions [16]. According to a study conducted by Carolyne Pehora et al., there are insufficient data to determine the efficacy of dexamethasone as an adjuvant to peripheral nerve block in lower limb surgeries. However, their study did find that dexamethasone used as an adjuvant to peripheral nerve block in upper limb surgeries may prolong the duration of sensory block and reduce postoperative pain and opioid consumption [17]. A meta-analysis by Chang Xiong and colleagues found that administering dexmedetomidine (Dexm) and dexamethasone (Dexa) through the perineural route provided the same duration of pain relief. The analysis showed that there was no significant difference between the effectiveness of the two drugs [18]. When dexmedetomidine or dexamethasone are added to 0.5% ropivacaine in a supraclavicular brachial plexus (SCBP) block, it enhances the quality of the block and reduces the need for pain relief for only 24 h. This improvement occurs without any negative side effects [19]. According to a study conducted by Braito et al., administering continuous wound infiltration (CWI) of ropivacaine 2 mg/mL at a rate of 2 mL/h for 24 h following hallux valgus surgery did not decrease postoperative pain levels for inpatients [20]. Levobupivacaine is more effective than ropivacaine in reducing postoperative pain for mini-abdominoplasty, according to a study by Kakagia et al. [21]. The use of ropivacaine for wound infiltration during laparoscopic colorectal resections does not provide substantial relief for pain management or prevent wound hyperalgesia [22]. Based on our study, it was observed that the R + DEX and R + DXM + DEXD groups took a longer time to recover from anesthesia as compared to the R and R + DXM groups. This is likely due to the low dosage of dexmedetomidine that entered the bloodstream. Our study did not confirm our hypothesis, and we found no evidence that adding dexamethasone and dexmedetomidine to local anesthetics during TLIF surgery could prolong the analgesic effect or enhance its efficacy. A study conducted by Weerasak Singhatanadgige and colleagues involved a randomized, controlled, double-blind trial. The results indicated that administering multimodal injections did not result in significant pain reduction. The VAS score differences did not exceed the minimum clinically important difference (MCID). The control group received a bupivacaine injection for wound infiltration after the surgical procedure [6]. Wound infiltration had no significant effect, but certain analgesic interventions could reduce opioid use. Insufficient evidence prevents a definitive recommendation for analgesic treatment after spinal fusion surgery [23]. Steroids' effectiveness compared to other options for pain relief is unclear. Local anesthetics are less effective when combined with tramadol than tramadol alone. The effectiveness of local infiltrates for relieving pain in lumbar surgery patients is inconclusive due to inconsistent methodology and insufficient data [24]. To summarize, while there are a few studies that support our findings, they are limited in number. In order to determine the best treatment for reducing postoperative pain and opioid use, and to avoid negative effects from local anesthetics in major spine surgeries, it is essential to conduct randomized, controlled trials on the use of dexamethasone and dexmedetomidine as adjuvants to local anesthetics.

## Limitations

Although our study design was randomized and controlled, no data were included regarding as severity of disease pathology, surgical approach (minimally invasive vs. open vs. endoscopic vs. luminal) and complexity of surgical procedure. A larger study population will be needed to evaluate safety and cost-effectiveness and synergistic effects of these drugs in different combinations.

## Conclusions

In our study, we did not find a significant reduction in postoperative pain or opioid consumption when adding dexamethasone and dexmedetomidine to ropivacaine for wound infiltration in transforaminal lumbar interbody fusion (TLIF). Additionally, this combination may produce some side effects. We recommend that this approach should not be used routinely in clinical practice.

## Data Availability

Researchers who make a valid request to the corresponding author will be given access to the data.

## References

[1] Rajan S, Devarajan J, Krishnaney A, George A, Rasouli JJ, Avitsian R (2022). Opioid alternatives in spine surgery: a narrative review. J Neurosurg Anesthesiol.

[2] Kurd MF, Kreitz T, Schroeder G, Vaccaro AR (2017). The role of multimodal analgesia in spine surgery. J Am Acad Orthop Surg.

[3] Woolf CJ, Chong MS (1993). Preemptive analgesia–treating postoperative pain by preventing the establishment of central sensitization. Anesth Analg.

[4] Bai JW, An D, Perlas A, Chan V (2020). Adjuncts to local anesthetic wound infiltration for postoperative analgesia: a systematic review. Reg Anesth Pain Med.

[5] Desai N, Kirkham KR, Albrecht E (2021). Local anaesthetic adjuncts for peripheral regional anaesthesia: a narrative review. Anaesthesia.

[6] Singhatanadgige W, Chancharoenchai T, Honsawek S, Kotheeranurak V, Tanavalee C, Limthongkul W (2020). No difference in pain after spine surgery with local wound filtration of morphine and ketorolac: a randomized controlled trial. Clin Orthop Relat Res.

[7] Zhang P, Liu S, Zhu J, Rao Z, Liu C. Dexamethasone and dexmedetomidine as adjuvants to local anesthetic mixture in intercostal nerve block for thoracoscopic pneumonectomy: a prospective randomized study. Regional anesthesia and pain medicine. 2019. 10.1136/rapm-2018-100221 31399540

[8] Lirk P, Hollmann MW, Strichartz G (2018). The science of local anesthesia: basic research, clinical application, and future directions. Anesth Analg.

[9] Mitra S, Purohit S, Sharma M (2017). Postoperative analgesia after wound infiltration with tramadol and dexmedetomidine as an adjuvant to ropivacaine for lumbar discectomies: a randomized-controlled clinical trial. J Neurosurg Anesthesiol.

[10] Bhardwaj S, Devgan S, Sood D, Katyal S (2017). Comparison of local wound infiltration with ropivacaine alone or ropivacaine plus dexmedetomidine for postoperative pain relief after lower segment cesarean section. Anesth Essays Res.

[11] Vallapu S, Panda NB, Samagh N, Bharti N (2018). Efficacy of dexmedetomidine as an adjuvant to local anesthetic agent in scalp block and scalp infiltration to control postcraniotomy pain: a double-blind randomized trial. J Neurosci Rural Pract.

[12] Li J, Yang JS, Dong BH, Ye JM (2019). The effect of dexmedetomidine added to preemptive ropivacaine infiltration on postoperative pain after lumbar fusion surgery: a randomized controlled trial. Spine.

[13] Mohamed SA, Sayed DM, El Sherif FA, Abd El-Rahman AM (2018). Effect of local wound infiltration with ketamine versus dexmedetomidine on postoperative pain and stress after abdominal hysterectomy, a randomized trial. Eur J Pain.

[14] Evaristo-Méndez G, García de Alba-García JE, Sahagún-Flores JE, Ventura-Sauceda FA, Méndez-Ibarra JU, Sepúlveda-Castro RR (2013). Analgesic efficacy of the incisional infiltration of ropivacaine vs ropivacaine with dexamethasone in the elective laparoscopic cholecystectomy. Cir Cir.

[15] Choi S, Rodseth R, McCartney CJ (2014). Effects of dexamethasone as a local anaesthetic adjuvant for brachial plexus block: a systematic review and meta-analysis of randomized trials. Br J Anaesth.

[16] Jones JS, Cotugno RE, Singhal NR, Soares N, Semenova J, Nebar S (2014). Evaluation of dexmedetomidine and postoperative pain management in patients with adolescent idiopathic scoliosis: conclusions based on a retrospective study at a tertiary pediatric hospital. Pediatr Crit Care Med J Soc Crit Care Med World Feder Pediatr Intens Crit Care Soc.

[17] Pehora C, Pearson AM, Kaushal A, Crawford MW, Johnston B (2017). Dexamethasone as an adjuvant to peripheral nerve block. Cochr Database Syst Rev.

[18] Xiong C, Han CP, Zhao D, Tang ZH, Zhang YF, Wang J (2021). Comparing the effects of dexmedetomidine and dexamethasone as perineural adjuvants on peripheral nerve block: a PRISMA-compliant systematic review and meta-analysis. Medicine.

[19] Singh N, Gupta S, Kathuria S (2020). Dexmedetomidine vs dexamethasone as an adjuvant to 0.5% ropivacaine in ultrasound-guided supraclavicular brachial plexus block. J Anaesthesiol Clin Pharmacol.

[20] Braito M, Dammerer D, Schlager A, Wansch J, Linhart C, Biedermann R (2018). Continuous wound infiltration after hallux valgus surgery. Foot Ankle Int.

[21] Kakagia DD, Fotiadis S, Tripsiannis G, Tsoutsos D (2007). Postoperative analgesic effect of locally infiltrated levobupivacaine in fleur-de-Lys abdominoplasty. Aesthetic Plast Surg.

[22] Beaussier M, Parc Y, Guechot J, Cachanado M, Rousseau A, Lescot T (2018). Ropivacaine preperitoneal wound infusion for pain relief and prevention of incisional hyperalgesia after laparoscopic colorectal surgery: a randomized, triple-arm, double-blind controlled evaluation vs intravenous lidocaine infusion, the CATCH study. Color Dis Off J Assoc Coloproctol Great Br Irel.

[23] Geisler A, Zachodnik J, Køppen K, Chakari R, Bech-Azeddine R (2022). Postoperative pain treatment after spinal fusion surgery: a systematic review with meta-analyses and trial sequential analyses. Pain Rep.

[24] Tsaousi G, Tsitsopoulos P, Pourzitaki C, Palaska E, Badenes R, Bilotta F (2021). Analgesic efficacy and safety of local infiltration following lumbar decompression surgery: a systematic review of randomized controlled trials. J Clin Med.

